# Adherence to antiretroviral treatment by adults in a rural area of Botswana

**DOI:** 10.4102/curationis.v38i1.1255

**Published:** 2015-05-29

**Authors:** Valerie J. Ehlers, Emmanuel T. Tshisuyi

**Affiliations:** 1 Department of Health Studies, University of South Africa, South Africa

## Abstract

**Background:** As antiretroviral therapy (ART) is becoming increasingly available to people in developing countries, ART adherence challenges assume ever greater significance. Often underlying treatment failure is the fact that suboptimal adherence to ART is the strongest predictor of failure to achieve viral suppression below the level of detection.

**Objectives:** The study's main objective was to identify factors affecting ART adherence levels, as well as the impact on immunologic and virologic responses in adult patients in one rural district in Botswana.

**Methods:** A cross-sectional quantitative survey, was used. Structured interviews were conducted with 300 ART patients between November 2011 and February 2012. Data were analysed, then presented in charts, graphs and frequency tables.

**Results:** The prevalence of non-adherence to ART was 14.0%. Motivators of good adherence included disclosure of HIV-positive status to more than one person, frequent adherence counselling, self-efficacy for adherence to ART, positive interactions between patients and healthcare providers; and using adherence partners. Barriers to adherence were forgetfulness, transportation costs to and from the clinic, time away from work and side-effects. There was a strong positive correlation between adherence, CD4 counts and viral load. Adherence was closely tied to immunologic and virologic improvements. Respondents with poor adherence were likely to have unsuppressed viral loads (OR 12.98, 95% CI 4.9–34).

**Conclusion:** Adherence to ART is closely tied to virologic, immunologic, and clinical outcomes. Increases in adherence levels resulted in significant improvements in these outcomes. Near perfect adherence, however, is required to maximise the likelihood of long-term clinical success, which could pose challenges to many ART patients, especially in resource-limited rural settings.

## Introduction

Anti-retroviral therapy (ART) prolongs the lives of people living with HIV and Acquired Immune Deficiency Syndrome (AIDS) and enhances their well-being (Steel, Nwokike & Joshi 2007). However, ART is not a cure and it must be taken every day for the rest of these patients’ lives. As ART is becoming increasingly available and affordable to eligible people in developing countries, more attention is focused on issues related to its rational use, as well as to ART adherence, which is a key element for reducing the spread of drug-resistant HIV (Steel *et al.* 2007).

### Problem statement

#### Background information

Since 2002, the government of Botswana has provided ART free of charge to all citizens of Botswana who qualify for treatment. The ART programme aims to suppress viral replication, restore the person's immune response, stop or at least delay the progression of the disease, reduce mortality and enhance the person's quality of life. However, there are concerns that the ART adherence levels might be suboptimal (below 95%), with potentially serious consequences (World Health Organization [WHO] 2006), including the development of HIV strains that are resistant to first-line antiretroviral drugs (ARVs). Second- and third-line ART regimens, which must be used in cases of ARV-resistant strains of HIV, are much more expensive than the first-line ARVs and are more likely to produce side-effects. According to the WHO (2005:2), ART adherence rates of at least 95% are required for optimal viral suppression and treatment success.

To achieve effective treatment outcomes and realise the benefits of ART, strict adherence to treatment instructions are critical. Adherence rates of 95% or more are required for optimum virologic suppression (Jani 2004; Poppa *et al.* 2003; Simoni *et al.* 2003). Suboptimal adherence to ART fails to achieve viral suppression below the level of detection (WHO 2006). Therefore, this study sought to determine factors that influence ART adherence amongst patients attending one clinic in Botswana.

#### Aim of the study

The overall aim of this study was to identify factors affecting ART adherence levels and the impact on immunologic and virologic responses in adult patients in one rural district in Botswana and, further, to use this knowledge to enhance the ART adherence rate amongst adult patients at the participating clinic, other clinics in Botswana and other sub-Saharan African (SSA) countries.

#### Research objectives

Specific objectives of this study were to:

- Identify factors influencing patients’ adherence levels to ART.
- Determine the association between ART adherence levels and immunologic and virologic responses.
- Recommend possible interventions in order to improve ART adherence levels.

#### Assumptions on which the study was based

The assumptions on which the study was based are as follows:

- ART adherence would increase CD4 cell counts and suppression of viral loads (VL).
- Adherence could be measured by regular collection of ARVs from the pharmacy.

The patients who collected their refills regularly and maintained high adherence rates, were expected to show immunologic recovery and virologic suppression as reflected in their increased CD4 cell counts and undetectable VL, respectively.

The study used routinely collected data recorded on patients’ medical records and pharmacy refill charts.

### Definitions of key terms and/or concepts

**Adherence** refers to the ability of the person living with HIV to be involved in choosing, starting, managing and maintaining a given therapeutic combination medication regimen in order to control HIV replication and improve immune function (Jani 2004). The effective use of ARVs requires an adherence level of at least 95%. In this study, adherence is measured as the regular and consistent collection of ARVs from the pharmacy. Adherence to ART should help to improve the patient's quality of life as his or her VL should decline and his or her CD4 counts should increase if the treatment is effective.

**Adult patient** implies any patient aged 21 years and older receiving ART at the participating clinic. The age of 21 was accepted as this is the age where persons can legally give sole consent to participate in a study in Botswana.

**ARVs** are medications used to treat HIV. There are different classes of ARVs and they act at different stages of the HIV life cycle (WHO 2006).

**ART** is the standard treatment consisting of at least three ARVs to maximally suppress the HIV and stop the progression of AIDS (WHO 2006).

**CD4 cells** are immune system cells which fight infections. The number of CD4 cells decreases when a person suffers from HIV, making him/her susceptible to opportunistic infections. CD4 cell counts provide a useful and reliable monitoring test commonly used as a surrogate for ART adherence (Poppa *et al.* 2003:58). It is also used to determine the stage of the disease and monitor response to therapy. The normal CD4 cell count is about 1000 cells/mm^3^ (WHO 2006). Treatment commences when the CD4 count is 250 cells/mm^3^ or less in Botswana (Poppa *et al.* 2003). CD4 cell counts increase with adherence to ART, whilst it decreases with non-adherence and/or ARV-resistant HIV strains (Poppa *et al.* 2003).

**VL** describes the amount of HIV in the blood, measured by the number of copies of HIV RNA per millilitre (copies/mL). The VL should be below the level of detection (less than 400 copies/mL) by six months after starting ART (Ministry of Health, Botswana [MOH] 2005), provided the patient adheres to the prescribed ART regimen and responds to the ARVs.

## Research methods and design

### Design

A cross-sectional survey, using structured interview schedules to collect data, was used in this study. The study was conducted at the Infectious Disease Control Centre (IDCC) clinic located at the district hospital where patients with HIV are reviewed every working day and put on ART.

### Population

The study population for this study comprised adult patients who had been on ART at the participating clinic for at least six months by 31 October 2011. Structured interviews were conducted with 300 ART patients between November 2011 and February 2012.

Eligible respondents were selected using systematic sampling during routine consultations until the required sample size of 300 was obtained.

### Data collection instrument

The structured interview schedule, comprising mostly open-ended items but including a few closed-ended questions, was designed specifically for this study. A pilot study was conducted by interviewing 10 patients, who were excluded from participation in the final study. This enabled the two interviewers to identify any potential comprehension and recording problems; and to estimate the time needed to complete each interview. Interviews lasted 22–40 minutes.

The different sections of the interview schedule sought information about the respondents’ demographic variables, health-related aspects, counselling experiences, adherence levels, remembering to take their pills regularly, disclosure issues, side-effects of ARVs and social issues which could influence their adherence levels.

The instrument's content and construct validity were evaluated by five healthcare professionals who provided ART services in Botswana. The reliability of the structured interview schedule was determined by calculating Cronbach's Alpha coefficients. These ranged from 0.79 to 0.85 for different subsections of the instrument, which was deemed acceptable (Burns & Grove 2001:399) for a newly-developed instrument.

### Data collection

The interviews were conducted in a private room in the clinic. Each respondent's informed consent was obtained before the interview commenced. The interviewers asked the same questions in the same sequence during each interview and recorded every response. Responses to open-ended questions were recorded verbatim. The first author was available if any interviewer required his assistance. Each respondent's pharmacy refill records, CD4 count and VL were recorded from his/her patient file from 01 January 2011 until the date of his/her interview.

### Data analysis

The *p*-value was set at < 0.05 for statistical significance. Associations between social factors and adherence were determined using Chi-squares or Fisher's exact test, where expected values were > 5. The outcome measure of adherence was based on the pharmacy refill data, the pill count and the self-report, and the correlation of these measures to the changes in the patient's CD4 cell count and the VL result.

## Results

Some biographic data will be presented initially so that the rest of the results can be contextualised against this background knowledge. The results will address patients’ adherence levels, the correlation between patients’ adherence levels and the CD4, as well as their VL counts and factors affecting patients’ ART adherence levels.

### Biographic data

The data summarised in Table 1 indicate that more women (74.3%; *n* = 223) than men (25.7%; *n* = 77) were interviewed. Of the 300 respondents, 65.0% (*n* = 195) had never been married, 15.3% (*n* = 46) were married, 14.0% (*n* = 42) were cohabiting and 5.7% (*n* = 17) were divorced, widowed or separated. Only 9.0% (*n* = 27) of the respondents had attained tertiary and 45.3% (*n* = 135) secondary education levels. This implies that 45.7% (*n* = 137) of the respondents, with no or primary level education, might have encountered difficulties with regard to understanding the nature of HIV, AIDS and the dire necessity to adhere strictly to the ART regimens. However, this possibility was not specifically tested in this study. Of the respondents, 56.7% (*n* = 170) had no monthly income, 16.0% (*n* = 48) earned less than $100 and only 7.3% (*n* = 22) earned $300 or more per month.

**TABLE 1:** Sociodemographic characteristics of the respondents (*N* = 300).

Variable	Number (N = 300)	Percentage
**Gender**		
Female	223	74.3
Male	77	25.7
**Age (years)**		
21–30	54	18.0
31–40	118	39.3
41–50	73	24.3
51–60	39	13.0
> 60	16	5.3
**Marital status**		
Never married	195	65.0
Married	46	15.3
Cohabiting	42	14.0
Divorced/separated/widowed	17	5.7
**Education status**		
None	44	14.7
Primary	93	31.0
Secondary	136	45.3
Tertiary	27	9.0
**Employment status**		
Employed	142	47.3
Unemployed	158	52.7
**Income (Pula)**		
No income	170	56.7
< 1000	48	16.0
1000–1999	43	14.3
2000–2999	17	5.7
≥ 3000	22	7.3

### Adherence to antiretrovirals

The mean ART adherence, measured by pill counts, was 97.6%, with a standard deviation of 3.7 and a median adherence level of 99.0%. As many as 86.0% (*n* = 258) of the respondents had optimal average adherence rates of at least 95.0% for four days preceding the interviews. However, 47.0% (*n* = 141) of the respondents’ clinic files indicated that they had missed pharmacy refill appointments and 32.7% (*n* = 98) sometimes took more than the prescribed number of pills. Forty-seven per cent (*n* = 141) of the respondents encountered income losses whilst attending ART clinics.

Of the 223 female respondents, 197 (88.3%) had a high level of adherence and 62 (80.5%) out of 77 male respondents also had high adherence levels. There was, however, no significant association between gender and adherence (χ^2^ = 2.97; *p* = 0.085). Although the 54 younger respondents (aged 21–30) and the 16 who were older than 60, had lower adherence levels of 83.3% and 81.3%, respectively; no statistically-significant trend was observed (χ^2^ = 1.11; *p* = 0.085) between age and adherence level.

### Important factors influencing patients’ antiretroviral therapy adherence levels

The three most common reasons, reported by 57 respondents, for missing ART doses were forgetfulness (6.3%; *n* = 19), running out of pills (4.0%; *n* = 12) and depression (3.3%; *n* = 10), but nobody mentioned pill burden.

Of the 141 (47.0%) respondents who lost income whilst attending ART clinics, some did so as a result of taking time off work, or forfeiting part of their income because of ART clinic attendance. Some of the suggestions provided by these 141 respondents to help them to improve their adherence levels without incurring losses of income, included:

- Employees should not lose payment for attending the ART clinics (37.6%; *n* = 53).
- Services should be provided closer to respondents’ homes/jobs (28.4%; *n* = 40).
- Transport to and from ART clinics should be provided for patients (22.0%; *n* = 31).
- Jobs should be created for patients on ART (12.2%; *n* = 17).

### Respondents’ adherence levels correlated with their CD4 counts and viral loads

Respondents (70.7%; *n* = 212) who had been on ART for more than 24 months, had an average adherence level of 88.4% which was higher than that of 75% which was reported for patients on treatment for 6–12 months. Only 52.7% (*n* = 158) of the respondents had recorded baseline CD4 counts ranging from 51 to 100 cells/µL, 35.7% (*n* = 107) equal to or less than 50 cells/µL and 11.6% (*n* = 35) had baseline CD4 counts greater than 100 cells/µL.

The majority of the respondents (87.3%; *n* = 262) had current CD4 counts greater than 200 cells/µL, indicating the success of treatment. Respondents’ current CD4 cell counts were marginally associated with ART adherence (χ^2^ = 3.99; *p* = 0.046). Low baseline CD4 cell counts (less than 100 cells/µL) were associated with higher optimal adherence levels to ART compared with their counterparts who had baseline CD4 cell counts of more than 100 cells/µL.

The majority of respondents (93.3%; *n* = 280) had undetectable VL counts. Amongst the 20 respondents who were *not* virologically suppressed, 12 (60.0%) had suboptimal ART adherence; but 29 (10.4%) respondents *with* suppressed VL also had suboptimal ART adherence levels. Of the respondents, only 9.3% (*n* = 28) were on second-line and 1.7% (*n* = 5) on third-line regimens. Changed ART regimens were because of: VL (37.7%; *n* = 20); drug toxicities (28.3%; *n* = 15); clinical failures (7.5%, *n* = 4); immunological failures (5.7%; *n* = 3); and pregnancies or changed treatment guidelines (15.1%; *n* = 8).

### Reasons for missing antiretroviral therapy doses

Side-effects experienced by the respondents included dizziness (30.3%; *n* = 91), headaches (27.3%; *n* = 82), nausea (23.0%; *n* = 69), skin/nail discolouration (18.7%; *n* = 56), depression (16.7%; *n* = 50), skin rashes (15.3%; *n* = 46), vomiting (14.3%; *n* = 43) and diarrhoea (7.3%; *n* = 22). Only 32 (10.7%) respondents reported that side-effects affected their daily activities.

The major reasons, reported by respondents, for missing ART doses were: forgetfulness (6.3%; *n* = 19), running out of pills (4.0%; *n* = 12) and depression (3.3%; *n* = 10). The most common strategies for remembering to take ARVs included: putting the pills in a visible place (90.0%; *n* = 270), setting alarms (79.3%; *n* = 238) and taking pills along when going away from home (77.0%; *n* = 231).

### Adherence and social aspects

Adherence partners included respondents’ mothers (20.0%; *n* = 60), children (17.7%; *n* = 53), spouses (16.0%; *n* = 48), brothers or sisters (13.3%; *n* = 40), friends (9.3%; *n* = 28) and fathers (0.7%; *n* = 2). Almost all respondents (99.7%; *n* = 299) were counselled before commencing ART. However, 58.0% (*n* = 174) reported that they were counselled once only, whereas 42.0% (*n* = 126) had been counselled more than once. No significant association existed between counselling and ART adherence (*p* = 0.151).

Sixty-three per cent (*n* = 189) of the respondents had disclosed their HIV-positive status to five or more people and their adherence level of 89.0% (95% CI 84–93, *p* < 0.005) was significantly higher than that of respondents who had disclosed only to one person (8.7%, *n* = 26), with an adherence level of 65.0% (95% CI 44–83). The respondents had disclosed their HIV-positive status to grandparents and to their parents-in-law (61.0%; *n* = 183), mothers (35.3%; *n* = 106), spouses or partners (26.0%; *n* = 78), friends (24.7%; *n* = 74), brothers and/or sisters (16.3%; *n* = 49), fathers (13.0; *n* = 39), sons and/or daughters (12.0%; *n* = 36). Fifty-five per cent (*n* = 166) reported that they disclosed their HIV-positive status on the same day that the diagnosis had been made, 11.0% (*n* = 33) disclosed within one week, 7.0% (*n* = 21) a week after the diagnosis, 17.7% (*n* = 53) within one to 12 months and 9.0% (*n* = 27) more than 12 months after learning their HIV-positive status.

Low levels of perceived stigma were reported. Only 20.0% (*n* = 60) of the respondents were worried about being shunned by the community, 21.0% (*n* = 63) feared being teased or insulted, whilst 18.0% (*n* = 54) were worried about losing the respect of their family members.

Most respondents (74.7%; *n* = 224) reported that they were able to meet the clinic staff (medical doctors and professional nurses) whenever they needed help, 78.0% (*n* = 234) reported that clinic staff understood the patients’ difficulties and (93.3%; *n* = 280) reported that the clinic staff encouraged patients to take their ARVs correctly.

Only 2.0% (*n* = 6) of the respondents had visited traditional healers since taking ARVs and 1.7% (*n* = 5) used traditional medicines together with ARVs. Almost all respondents (98.0%; *n* = 294) reportedly did not believe in traditional medicines.

Sixteen per cent of the respondents (*n* = 48) admitted to alcohol consumption and 10.0% (*n* = 31) to cigarette smoking. As many as 71.7% (*n* = 215) of the respondents indicated that they had only one sex partner, 27.0% (*n* = 81) had no sex partner, whilst 1.3% (*n* = 4) had two or more sex partners. Of those who had sex partners, 52.9.0% (*n* = 116) reported having had the same partner for more than five years, 24.2% (*n* = 53) for 2–5 years and 23.3% (*n* = 51) for one year or less. Of these 219 respondents’ sex partners, 45.2% (*n* = 99) were HIV-positive, 39.3%% (*n* = 86) were HIV negative and 16.0% (*n* = 37) had an unknown HIV status.

## Ethical considerations

Permission to carry out the study was granted by the University of South Africa as this study comprised part of the first author's obligations to be fulfilled for his Master's degree in Public Health (MPH). The MOH and the participating healthcare institution's management also gave permission for the study to be conducted at the participating clinic.

Signed informed consent was obtained prior to each interview. Participation was voluntary and refusal to participate in the study had no effect whatsoever. No respondent was coerced to answer any specific question. Every respondent was informed that he/she could terminate the interview at any stage. If any respondent should have terminated an interview, another respondent would be requested to consider participating in the study. Although no respondent terminated any interview, they did not reply to all questions. No remuneration was given. Every signed consent form was sealed in an envelope and placed in a container. Each anonymously-completed interview schedule was placed in another container so that no one could match any completed interview schedule with a specific signed consent form. The first author collected the signed consent forms and completed interview schedules at regular intervals and kept these documents securely locked up. The data entered onto the first author's computer could only be accessed by a secure password. Only the first author and the statistician had access to this data and to the completed interview schedules. Every respondent was assured about the anonymity and confidentiality of the data and was informed that a copy of the research report could be requested from the first author should they wish to do so.

## Trustworthiness

### Reliability and validity

Five healthcare providers, who had undergone training in Botswana's HIV treatment protocols and who were working in HIV clinics, evaluated the items in the structured interview schedules. These five experts agreed that all items were relevant to ART adherence issues, amounting to face validity. Furthermore, they also agreed that the instrument's items addressed constructs in and the content of Botswana's HIV treatment guidelines (MOH 2005), amounting to content and construct validity.

Reliability of the instrument was acceptable because the 10 interviews conducted during the pretesting phase produced information similar to that obtained from 300 interviews conducted to collect data for the actual study. The Cronbach alpha score of 0.85 was accepted as indicating that the instrument's items related to ART adherence issues.

## Discussion

Of the 300 respondents, 86.0% (*n* = 259) reported optimal ART adherence (≥ 95.0%), higher than findings reported by other researchers (77.0% by Kgatlwane *et al.* 2006; 54.0% by Weiser *et al.* 2003; and 83.0% by Nwokike 2003); possibly showing an improvement in adherence levels by patients on ART in Botswana. However, the ultimate goal is to increase the adherence level to equal to or greater than 95.0% at individual level as well as at the population level (Weiser *et al.* 2003).

In this study, it was reported that costs incurred through transport and lost wages to visit ART clinics were major obstacles that had a negative impact on optimal adherence to ART. In another Botswana study (Kip, Ehlers & Van der Wal 2009), 45.0% of the respondents also reported the same burden of payment for their transport to ART clinics. Similar financial concerns were reported in Uganda, Tanzania and Botswana (Hardon *et al.* 2007:660).

In this study, side-effects of ART, such as dizziness, headaches and nausea, were reported – similar to those reported by Kip *et al.* (2009:149). However, no association between side-effects of ARVs and ART adherence levels was found. This finding is consistent with reports from a previous study conducted in Côte d’Ivoire (Diabaté, Alary & Koffi 2007:1801). The most common reasons for missing ARVs were: forgetfulness, running out of pills and depression. No respondent complained of pill burden. Findings from a similar study conducted in Botswana (Kgatlwane *et al.* 2006), reported that the most common reasons for missing ARVs were forgetfulness, costs/logistics and work/home duties, also without any pill burden problem.

Common reminders for taking medicines were alarm clocks, putting pills in visible places and taking pills along when going away from home. Almost all of the respondents (99.7%; *n* = 299) were counselled prior to starting ART, along with their adherence partners. The use of family members and peers enhanced ART adherence, which is similar to findings from previous studies conducted in Botswana (Kgatlwane *et al.* 2006; Kip *et al.* 2009).

Most respondents (97.6%; *n* = 293) had high levels of confidence (self-efficacy) with regard to adherence to ART, similar to the findings of previous studies (Catz *et al.* 2000; Chesney 2000; Gifford *et al.* 2000; Godin *et al.* 2005; Kip *et al.* 2009; Wilson, Doxanakis & Fairley 2004).

Low levels of perceived stigma did not influence ART adherence levels, which is again consistent with findings from previous studies (Kgatlwane *et al.* 2006; Weiser *et al.* 2003). Patients who had disclosed their HIV-positive status to more than one person had higher adherence levels than those who disclosed to only one person. This finding was similar to that reported by Kip *et al.*’s (2009) study in Botswana.

Most respondents (93.3%; *n* = 280) were satisfied with the quality of ART care provided at the clinics. No social or cultural factors (such as alcohol consumption, cigarette smoking, HIV status and number of sexual partners or traditional medicines) had any significant association with ART adherence levels (*p* > 0.05) and could thus not be regarded as factors influencing patients’ ART adherence levels.

Respondents’ most recent CD4 cell counts were associated with ART adherence (χ^2^ = 3.99; *p* = 0.046). Most respondents (93.0%) had undetectable VL, but this was not the case for those with poor adherence levels (OR 12.98, 95% CI 4.9–34), as shown in Table 3.

**TABLE 2:** Social and cultural factors influencing antiretroviral therapy adherence levels.

Question	Answer	Frequency	Percent	-value (Fishersa or Chi-squareb)
Having visited a traditional healer since commencing ART	No	294	98.0	0.191a (ns)
	Yes	6	2.0	
Taking any traditional medicines concurrently with ARVs	No	295	98.3	0.139a (ns)
	Yes	5	1.7	
Considerations that traditional medicines are effective	No	294	98.0	0.191a (ns)
	Yes	6	2.0	
Drink alcohol	No	252	84.0	0.425a (ns)
	Yes	48	16.0	
Smoking cigarettes?	No	269	90.0	0.591a (ns)
	Yes	31	10.0	
Number of sexual partners	None	81	27.0	0.850a (ns)
	1	215	71.7	
	2	4	1.3	
Current sex partner	≤ 3 months	16	7.3	0.282b (ns)
	4–6 months	16	7.3	
	7–12 months	19	8.6	
	2–5 years	53	24.1	
	> 5 years	116	52.7	
HIV status of sex partner	Positive	99	33.0	0.546b (ns)
	Negative	86	28.7	
	Unknown	37	12.3	
	No response	78	26.0	

ART, antiretroviral therapy; ARVs, antiretroviral drugs; ns, not significant.

**TABLE 3:** Correlations between selected factors and adherence.

Variables	Age	CD4	VL copies	CD4 current	Adh avg
**Kendall's tau _b**					
Age: Correlation coefficient	1.000	-0.045	−0.115*	0.009	0.044
Significance (2-tailed)	-	0.296	0.027	0.844	0.351
*n*	300	300	300	299	300
CD4: Correlation coefficient	−0.045	1.000	−0.043	0.209**	0.042
Significance (2-tailed)	0.296	-	0.356	0.000	0.321
*n*	300	300	300	299	300
VL copies: Correlation coefficient	−0.115*	−0.043	1.000**	−0.197**	-0.246**
Significance (2-tailed)	0.027	0.356	-	0.000	0.000
*n*	300	300	300	299	300
CD4 current: Correlation coefficient	0.009	0.209**	−0.197**	1.000	0.022
Significance (2-tailed)	0.844	0.000	0.000	-	0.612
*n*	299	299	299	299	299
Adh Avg: Correlation coefficient	0.044	0.042	−0.246**	0.022	1.000
Significance (2-tailed)	0.351	0.321	0.000	0.612	-
*n*	300	300	300	299	300
**Spearman's rho**					
Age: Correlation coefficient	1.000	−0.061	−0.129*	0.012	0.055
Significance (2-tailed)	-	0.296	0.026	0.835	0.345
*n*	300	300	300	299	300
CD4: Correlation coefficient	−0.061	1.000	−0.054	0.310**	0.058
Significance (2-tailed)	0.296	-	0.352	0.000	0.314
*n*	300	300	300	299	300
VL copies: Correlation coefficient	−0.129*	-0.054	1.000	−0.243**	0.279**
Significance (2-tailed)	0.026	0.352	-	0.000	0.000
*n*	300	300	300	299	300
CD4 current: Correlation coefficient	0.012	0.310**	−0.243**	1.000	0.031
Significance (2-tailed)	0.835	0.000	0.000	-	0.593
*n*	299	299	299	299	299
Adh Avg: Correlation coefficient	0.055	0.058	−0.279**	0.031	1.000
Significance (2-tailed)	0.345	0.314	0.000	0.593	-
*n*	300	300	300	299	300

ART, antiretroviral therapy; CD4, CD4 count (when the patients commenced ART); CD4 current, the patients’ CD4 counts at the time of data collection; VL, viral load; *n*, frequency (number of respondents); adh avg, adherence average (to ART).

*, correlation is significant at the 0.05 level (2-tailed); **, correlation is significant at the 0.01 level (2-tailed)

There was no association between employment, loss of income, education status, marital status, monthly income, age and ART adherence levels.

## Limitations of the study

The study was conducted at one clinic where 300 ART patients were interviewed. All these patients lived within the catchment area of the clinic. This limits the generalisability of the findings. Interviews were only conducted with the patients and did not include their relatives and the healthcare providers at the clinic. These persons might have had different ideas about factors influencing the patients’ adherence levels to their ART. However, experiences of the patients themselves comprised the data for this study.

## Recommendations

In order to enhance patients’ ART adherence levels at the participating clinic, the following recommendations were made:

- Regular assessments of ART adherence should be done at the clinic and suboptimal adherence levels must be addressed so as to avoid virologic and immunologic failures as well as drug resistance.
- In order to reduce costs, patients on ART could be issued with three months’ supplies of ARVs instead of one month's supply when they come for review.
- Future research studies should involve patients from more than one clinic.
- Qualitative in-depth interviews should be conducted with patients, their close family members and healthcare providers in order to provide different insights into factors influencing patients’ ART adherence behaviours.

### Implications

Free supply of ARVs to eligible persons in Botswana does not ensure sufficiently high levels of ART adherence to both achieve and maintain VL suppression beyond detectable limits as well as increases in CD4 counts. Adherence might be enhanced if ARV supplies could be issued for three months at every clinic visit rather than for one month only.

## Conclusion

In conclusion, the findings of this study showed that motivators of ART adherence included disclosure of one's HIV-positive status to more than one person, self-efficacy and adherence partners. Barriers to ART adherence included forgetfulness, transportation costs to and from ART clinics, as well as income lost because of absence from work to visit ART clinics. This then implies that these aspects had a negative influence on the respondents’ adherence levels. In addition, there was a positive correlation between respondents’ ART adherence levels, increased CD4 counts and undetectable VLs.

## References

[CIT0001] Burns N. & Grove S.K., 2001, *The practice of nursing research: conduct, critique,* & *utilization*, 4th edn., W.B. Saunders, Philadelphia, PA.

[CIT0002] Catz S.L., Kelly J.A., Bogart L.M., Benotsch E.G. & McAuliffe T.L., 2000, ‘Patterns, correlates, and barriers to medication adherence among persons prescribed new treatments for HIV disease’, *Health Psychology* 19(2), 124–133. 10.1037/0278-6133.19.2.124 10762096

[CIT0003] Chesney M.A., 2000, ‘Factors affecting adherence to anti-retroviral therapy’, *Clinical Infectious Diseases* 30(Suppl 2), S171–S176. 10.1086/313849 10860902

[CIT0004] Diabaté S., Alary M. & Koffi C.K., 2007, ‘Determinants of adherence to highly active antiretroviral therapy among HIV-1-infected patients in Côte d’Ivoire’, *AIDS* 21(13), 1799–1803. 10.1097/QAD.0b013e3282a5667b 17690579

[CIT0005] Gifford A.L., Bormann J.E., Shively M.J., Wright B.C., Richman D.D. & Bozzette S.A., 2000, ‘Predictors of self-reported adherence and plasma HIV concentrations in patients on multidrug antiretroviral regimens’, *Journal of Acquired Immune Deficiency Syndromes* 23(5), 386–395. 10.1097/00126334-200004150-00005 10866231

[CIT0006] Godin G., Côté J., Naccache H., Lambert L.D. & Trottier S., 2005, ‘Prediction of adherence to antiretroviral therapy: A one-year longitudinal study’, *AIDS Care* 17(4), 493–504. 10.1080/09540120412331291715 16036235

[CIT0007] Hardon A.P., Akurut D., Comoro C., Ekezie C., Irunde H.F., Gerrits T. et al., 2007, ‘Hunger, waiting time and transport costs: Time to confront challenges to ART adherence in Africa’, *AIDS Care* 19(5), 658–665. 10.1080/09540120701244943 17505927

[CIT0008] Jani A.A., 2004, ‘Adherence to HIV treatment regimens: Recommendations for best practices’, viewed 17 July 2009, from http://www.apha.org/ppp/hiv [URL no longer valid].

[CIT0009] Kgatlwane J., Ogenyi R., Ekezie C., Madaki H.N., Moyo S. & Moroka T.M., 2006, ‘Factors that facilitate or constrain adherence to antiretroviral therapy among adults at four public health facilities in Botswana: A pre-intervention study’, in World Health Organization, *From access to adherence: The challenges of antiretroviral treatment* – *studies from Botswana, Tanzania and Uganda, 2006*, WHO, Geneva; pp. 75–164.

[CIT0010] Kip E., Ehlers V.J. & Van Der Wal D.M., 2009, ‘Patients’ adherence to anti-retroviral therapy in Botswana’, *Journal of Nursing Scholarship* 41(2), 149–157. 10.1111/j.1547-5069.2009.01266.x 19538699

[CIT0011] Ministry of Health (Botswana), 2005, *Botswana national HIV/AIDS treatment guidelines*, Government Printer, Gaborone.

[CIT0012] Nwokike J., 2003, ‘Baseline data and predictors of adherence to antiretroviral therapy in Maun General Hospital (MGH), Maun, Botswana’, *Antiviral Therapy* 8, S396.

[CIT0013] Poppa A., Davidson O., Deutsch J., Godfrey D., Fisher M., Head S. et al., 2003, ‘British HIV Association (BHIVA)/British Association for Sexual Health and HIV (BASHH) guidelines on provision of adherence support to individuals receiving antiretroviral therapy (2003)’, HIV Medicine 5(Suppl 2), 46–60. 10.1111/j.1468-1293.2004.00215.x 15239716

[CIT0014] Simoni J.M., Frick P.A., Pantalone D.W. & Turner B.J., 2003, ‘Anti-retroviral adherence interventions: A review of current literature and ongoing studies’, *Topics in HIV Medicine* 11(6), 185–197. 14724327

[CIT0015] Steel G., Nwokike J. & Joshi M.P., 2007, *Development of a multi-method tool to measure ART adherence in resource-constrained settings: The South Africa experience*, viewed 12 December 2010, from http://pdf.usaid.gov/pdf_docs/PNADK153.pdf

[CIT0016] Weiser S., Wolfe W., Bangsberg D., Thior I., Gilbert P., Makhema J. et al, 2003, ‘Barriers to anti-retroviral adherence for patients living with HIV infection and AIDS in Botswana’, *Journal of Acquired Immune Deficiency Syndromes* 34(3), 281–288. 10.1097/00126334-200311010-00004 14600572

[CIT0017] Wilson K.J., Doxanakis A. & Fairley C.K., 2004, ‘Predictors for non-adherence to antiretroviral therapy’, *Sexual Health*, 1(4), 251–257. 10.1071/SH04020 16335755

[CIT0018] World Health Organization, 2005, ‘Baseline data and predictors of adherence in patients on anti-retrovir al therapy in Maun general hospital, Botswana’, *Essential Drugs Monitor* 34, 12–36.

[CIT0019] World Health Organization, 2006, ‘From access to adherence: The challenges of antiretroviral treatment – studies from Botswana, Tanzania and Uganda’ viewed 07 October 2009, from http://apps.who.int/medicinedocs/en/d/Js13400e/

